# Banana Leaves Imagery Dataset

**DOI:** 10.1038/s41597-025-04456-4

**Published:** 2025-03-21

**Authors:** Neema Mduma, Christian Elinisa

**Affiliations:** Nelson Mandela African Institution of Science and Technology, P. O. Box 447, Tengeru, Arusha Tanzania

**Keywords:** Agriculture, Developing world

## Abstract

In this work, we present a dataset of banana leaf imagery, both with and without diseases. The dataset consists of 11,767 images, categorized as follows: 3,339 healthy images, 3,496 images of leaves affected by Black Sigatoka and 4,932 images of leaves affected by Fusarium Wilt Race 1. This data was collected to support machine learning diagnostics for disease detection. The data collection process involved farmers, researchers, agricultural experts and plant pathologists from the northern and southern highland regions of Tanzania. To ensure unbiased representation, farms were randomly selected from the Rungwe, Mbeya, Arumeru, and Arusha districts, based on the presence of banana crops and the targeted diseases. The dataset offers a comprehensive collection of images captured from November 2022 to January 2023, using a high-resolution smartphone camera across a wide geographical area. Researchers and developers can use this dataset to build machine learning solutions that automatically detect diseases in images, potentially enabling agricultural stakeholders, including farmers, to diagnose Fusarium Wilt Race 1 and Black Sigatoka early and take timely action.

## Background & Summary

Bananas are a staple food and cash crop for nearly 70 million farmers in Africa’s humid and sub-humid tropics^1^. The crop is, however, highly susceptible to diseases, particularly to Black Sigatoka and Fusarium Wilt Race 1^2^. These diseases can cause yield losses ranging from 30% to 100%, depending on the banana cultivar and the severity of the infection^3^. Therefore, effective disease management strategies are essential to protect banana crops and ensure food security in Africa. The current method of identifying and confirming banana diseases relies on visual inspection of leaves, stems and other affected plant parts. This approach has limitations such as delayed diagnosis beyond the point of recovery which can result in substantial disease management and control expenses and potentially significant or even complete crop losses.

Several researchers have demonstrated the potential of using machine learning tools for diagnosis of diseases in different crops such as tomatoes, cassavas, and bananas^4–6^. This approach has proven to assist agricultural stakeholders, including smallholder farmers and extension officers, in automatically identifying crop diseases as early as possible for proper and effective interventions. However, training machine learning models requires a large amount of data, and developing countries like Tanzania lack enough data to facilitate research activities in this field. To address this issue, the dataset of banana leaf images was published in the Harvard repository in 2022^7^. The dataset comprised 16,092 images, including 5,628 healthy leaves, 5,767 affected by Black Sigatoka and 4,697 showing symptoms of Fusarium Wilt Race 1, all gathered between February and July 2021. Although the dataset was extensive and included a broad range of instances, it did not fully represent data from all farming seasons. Recognizing this limitation, this article contributes to the agricultural sector by updating the dataset to include missing farming season data, a crucial component for accurate disease detection and analysis.

## Methods

### Collection of field data

Table 1 outlines the timeline for the imagery data acquisition process, which took place from November 22, 2022, to January 13, 2023, in Tanzania’s Northern and Southern Highlands regions. Imagery data of banana leaves were collected using a random sampling technique to select farms across four districts: Rungwe, Mbeya, Arumeru, and Arusha. A total of 51 farms participated in the study, with 14 farms in Rungwe, 11 in Mbeya, 12 in Arumeru and 14 in Arusha. These farms were chosen by agricultural extension officers and farmers’ representatives, focusing on areas with a high incidence of two diseases that significantly affect banana production.

**Table 1:** Timeline of the data acquisition process.

Process	Duration	Activity
Data collection	November 2022 to January 2023	Taking picture of banana leaves
Data annotation	January to February 2023	Labeling of the collected banana imagery data
Data preprocessing	February to March 2023	Cleaning, cropping and renaming of the banana imagery data

**Fig. 1:**
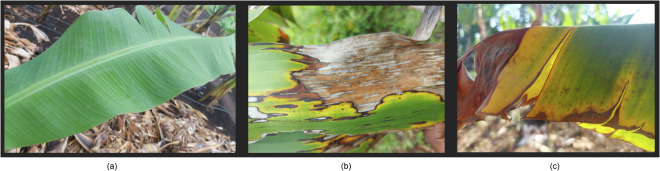
Sample of collected images (**a**) Healthy (**b**) Black Sigatoka (**c**) Fusarium Wilt Race 1.

Images were captured using a Samsung SM-A715F/DS smartphone camera equipped with the Open Data Kit tool, with a sample of these images presented in Fig. 1. The data collection process was carried out by farmers and researchers, under the supervision of experts from the International Institute of Tropical Agriculture (IITA) and the Nelson Mandela African Institution of Science and Technology (NM-AIST). To ensure accuracy and reliability, the collected data were thoroughly quality-checked by agricultural experts and plant pathologists.

### Data preprocessing

**Fig. 2:**
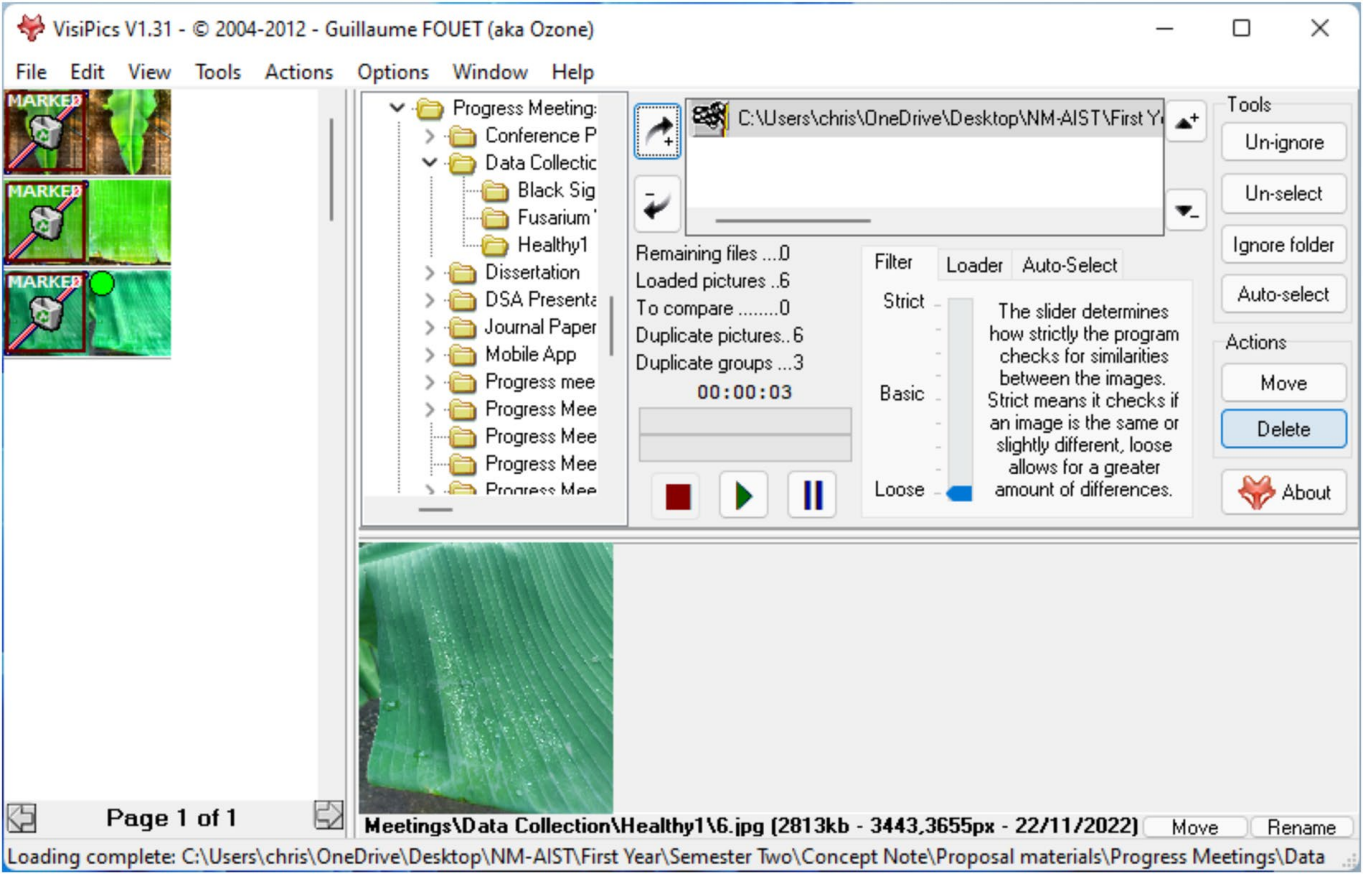
Method used for detecting and removing identical duplicate images.

The banana leaf images were preprocessed by cleaning, cropping, renaming and annotating. The images were manually cropped to remove extraneous background elements and emphasize the key features. VisiPics and Duplicate Photo Finder were used to check for duplicate images^8^. The selection of this software was based on its open-source nature and user-friendliness. VisiPics removed strictly identical images, while Duplicate Photo Finder utilized the “same picture” filter for image comparison. Figure 2 illustrates the use of VisiPics in detecting and deleting identical duplicates. It shows the process where VisiPics scans the image library, compares the visual content to identify exact matches and provides the user with an interface to review and remove these duplicates.

**Fig. 3:**
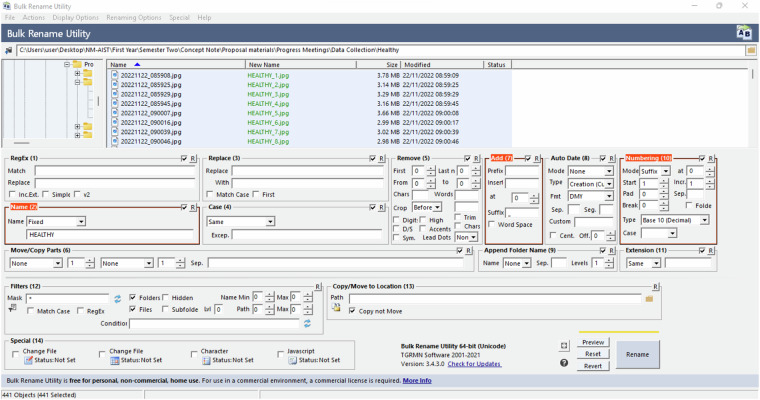
Method used to rename images.

The cleaned images were renamed to reflect the class names (HEALTHY, FUSARIUM WILT RACE 1, BLACK SIGATOKA), and the corresponding image number, i.e., HEALTHY_105.jpg. Bulk Rename Utility software was used to rename images for all classes as shown in Fig. 3. Also, images were labelled for classification and segmentation, among other computer vision tasks.

## Data Records

### Data file description

**Fig. 4:**
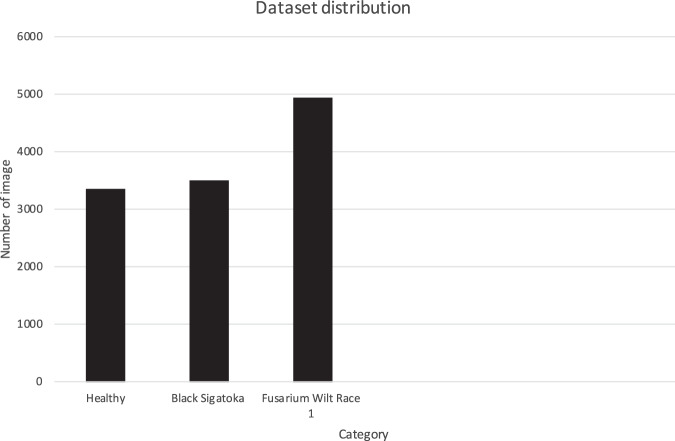
Distribution of the dataset.

This article presents the published dataset of banana leaf images which has been deposited in the Zenodo repository and published on February 23, 2023^9^. The dataset of 11,767 images in Joint Photographic Experts Group (JPEG) format was categorized into three classes: Healthy (3339 images), Black Sigatoka (3496 images) and Fusarium Wilt Race 1 (4932 images) as illustrated in Fig. 4.

**Fig. 5:**
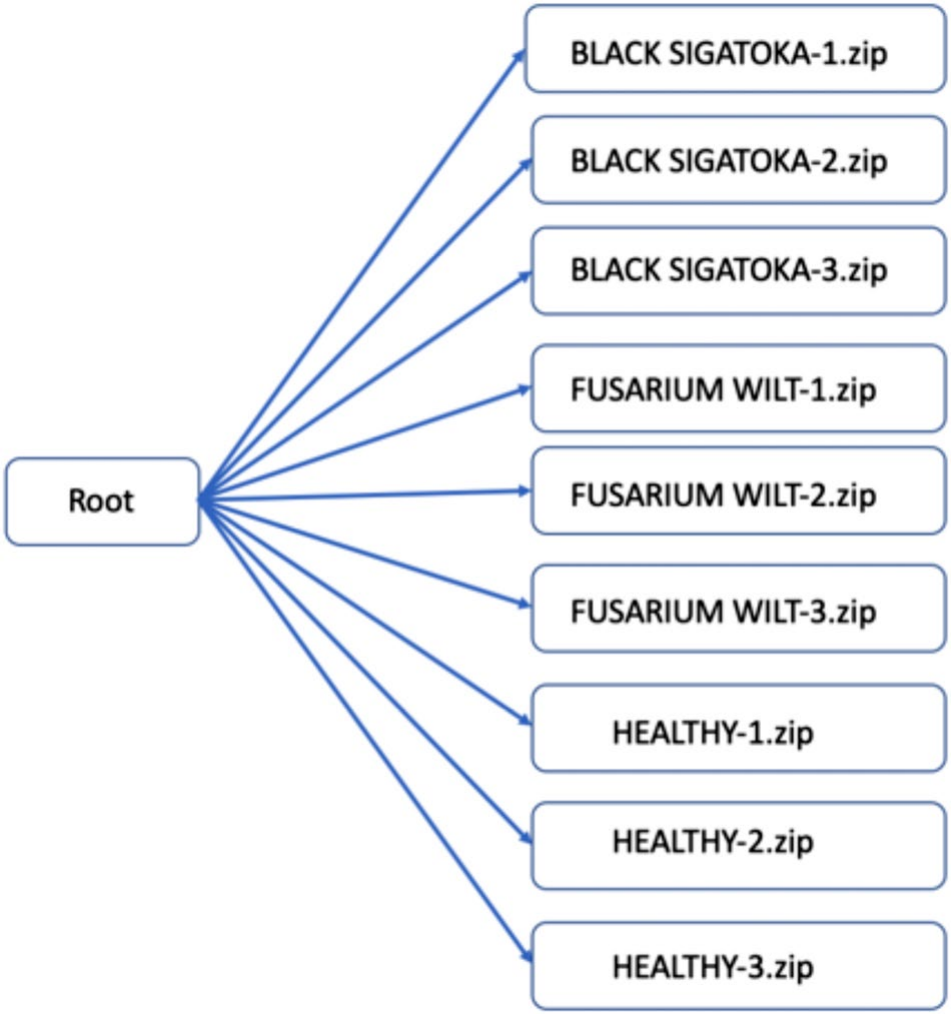
Data structure of the repository.

The data repository structure is presented in Fig. 5. In the repository, the root folder consists of nine separate zipped folders named to indicate the class name and folder number; three folders of Fusarium Wilt Race 1 (FUSARIUM WILT-1.zip, FUSARIUM WILT-2.zip, FUSARIUM WILT-3.zip), three folders of Black Sigatoka (BLACK SIGATOKA-1.zip, BLACK SIGATOKA-2.zip, BLACK SIGATOKA-3.zip) and three folders of healthy (HEALTHY-1.zip, HEALTHY-2.zip, HEALTHY-3.zip). The total size of the dataset is 34.3 GB, distributed as follows: BLACK SIGATOKA-1.zip (3.7 GB), BLACK SIGATOKA-2.zip (3.3 GB), BLACK SIGATOKA-3.zip (5.1 GB), FUSARIUM WILT-1.zip (3.1 GB), FUSARIUM WILT-2.zip (5.0 GB), FUSARIUM WILT-3.zip (7.2 GB), HEALTHY-1.zip (2.0 GB), HEALTHY-2.zip (2.3 GB) and HEALTHY-3.zip (2.6 GB).

## Technical Validation

The dataset contains clear and in focus images with sufficient resolution of 3480 × 3496 pixels in jpeg format. The study used Python scripts to perform the processing steps within a Conda virtual environment. This environment was configured with Python 3.9 and included the GoogleDriveDownloader 0.4 library for downloading datasets, along with Pandas 1.3.5 for data manipulation and analysis. Leveraging the freely available software, LabelMe was used to annotate images specifically for instance and semantic segmentation tasks. For instance, segmentation with Mask R-CNN, individual objects were identified by manually drawing irregular polygon masks around the Regions of Interest (ROIs) within the banana plant images. For semantic segmentation with U-Net, regions were segmented based on their semantic meaning. Each outlined ROI was then assigned a specific class label, providing crucial information for training both deep learning models.

The dataset focuses on banana imagery leaves, both healthy and those infected with two specific diseases, i.e., Fusarium Wilt Race 1 and Black Sigatoka, which significantly impact productivity in Tanzania. All classes are equally distributed to avoid data imbalance which may led to biasness. Images in this dataset were taken using a high-quality smartphone camera to avoid bias during model development. Future datasets can explore the possibility of incorporating other banana diseases apart from the two presented in this article.

## Data Availability

The data was preprocessed using Bulk Rename Utility: https://www.bulkrenameutility.co.uk for renaming the data, VisiPics: https://visipics.en.softonic.com for removing duplicates and LabelMe: https://github.com/labelmeai/labelme for data annotation.

## References

[1] FAO. Acting together against banana diseases in Africa from food and agriculture organizanion of United Nation. http://www.fao.org/agriculture/crops/news-eventsbulletins/detail/en/item/36259/icode/en/?no_cache=1 (2021).

[2] Sanga, S. L. *et al*. Mobile-based deep learning models for banana disease detection. *Engineering, Technology & Applied Science Research* **10**(3), 5674–5677, 10.48084/etasr.3452 (2020).

[3] ProMusa Vézina, A., & Van den Bergh, I. Black leaf streak. https://www.promusa.org/Black+leaf+streak (2020).

[4] Mkonyi, L. *et al*. Early identification of Tuta absoluta in tomato plants using deep learning. Scientific African 10.1016/j.sciaf.2020.e00590 (2020).

[5] Nabenda, J. N. *et al*. A dataset of necrotized cassava root cross-section images. Data in Brief 10.1016/j.dib.2020.106170 (2020). 10.1016/j.dib.2020.106170 PMC7452662 32904393

[6] Sanga, S. *et al*. Mobile-based deep learning models for banana diseases detection. The International Conference on Learning Representations (ICLR) https://arxiv.org/abs/2004.03718 (2020).

[7] Mduma, N., & Leo, J. Dataset of banana leaves and stem images for object detection, classification and segmentation: A case of Tanzania. Data in Brief 10.1016/j.dib.2023.109322 (2023). 10.1016/j.dib.2023.109322 PMC10333424 37441627

[8] Softonic. Software to detect and remove duplicate pictures. https://visipics.en.softonic.com (accessed January 02, 2024).

[9] Mduma, N. & Elinisa, C. Banana imagery dataset – Tanzania. *Zenodo*10.5281/zenodo.7670326 (2023).

